# Pre-Stroke Modified Rankin Scale: Evaluation of Validity, Prognostic Accuracy, and Association with Treatment

**DOI:** 10.3389/fneur.2017.00275

**Published:** 2017-06-13

**Authors:** Terence J. Quinn, Martin Taylor-Rowan, Aishah Coyte, Allan B. Clark, Stanley D. Musgrave, Anthony K. Metcalf, Diana J. Day, Max O. Bachmann, Elizabeth A. Warburton, John F. Potter, Phyo Kyaw Myint

**Affiliations:** ^1^Institute of Cardiovascular and Medical Sciences, University of Glasgow, Glasgow, United Kingdom; ^2^Norwich Medical School, University of East Anglia, Norwich, United Kingdom; ^3^Stroke Research Group, Norfolk and Norwich University Hospital, Norwich, United Kingdom; ^4^Lewin Stroke & Rehabilitation Unit, Addenbrooke’s Hospital, Cambridge, United Kingdom; ^5^Institute of Applied Health Sciences, University of Aberdeen, Aberdeen, United Kingdom

**Keywords:** modified Rankin scale, disability, stroke, outcome, complications, prognosis

## Abstract

**Background and purpose:**

The modified Rankin Scale (mRS) was designed to measure poststroke recovery but is often used to describe pre-stroke disability. We sought to evaluate three aspects of pre-stroke mRS: validity as a measure of pre-stroke disability; prognostic accuracy and association of pre-stroke mRS scores, and process of care.

**Methods:**

We used data from a large, UK clinical registry. For analysis of validity, we compared pre-stroke mRS against other markers of pre-stroke function (age, comorbidity index, care needs). For analysis of prognostic accuracy, we described univariable and multivariable models comparing pre-stroke mRS and other prognostic variables against a variety of outcomes (early and late mortality, length of stay, institutionalization, incident complications). Finally, we described association of pre-stroke mRS and components of evidence-based stroke care (early neuroimaging, admission to stroke unit, assessment of swallow).

**Results:**

We analyzed data of 2,491 stroke patients. Concurrent validity analyses suggested statistically significant, but modest correlations between pre-stroke mRS and chosen variables (rho >0.40; *p* < 0.0001 for all). Every point increase of pre-stroke mRS was associated with poorer outcomes for our prognostic variables (unadjusted *p* < 0.001). This association held when corrected for other covariates. For example, pre-stroke mRS 4–5 odds ratio (OR): 6.84 (95% CI: 4.24–11.03) for 1 year mortality compared to mRS 0 in adjusted model. There was a difference between pre-stroke mRS and treatment, with higher pre-stroke mRS more likely to receive evidence-based care.

**Conclusion:**

Results suggest that pre-stroke mRS has some concurrent validity and is a robust predictor of prognosis. This association is not explained by the influence of pre-stroke mRS on care pathways.

## Introduction

Disability and comorbidity are common in older adult populations. Demographic change will see increasing absolute numbers of older people with some degree of disability and comorbidity (1). Although stroke is a common cause of disability, many who experience stroke already have premorbid functional problems (2). To understand post stroke recovery and to plan realistic rehabilitation goals, it is important to have a method of describing pre-stroke disability.

As well as clinical utility, robust measures of pre-stroke function are also needed for research. Pre-stroke disability is often employed as exclusion criterion or case mix adjuster for trials and international registries (3, 4). This approach recognizes that even the best treatment is unlikely to improve function to better than the pre-stroke state. Pre-stroke disability may also have important prognostic utility, although data on this have been conflicting (5, 6). Many acute stroke risk stratification scales feature pre-stroke disability as a covariate (7, 8).

While the need to describe pre-stroke function is apparent, the method of achieving this is less certain. The modified Rankin scale (mRS) is a measure of global disability that is commonly used as a functional outcome for stroke studies (9). The mRS was designed to assess poststroke recovery levels and the wording of the original scale assumes a comparison with the pre-stroke state. The scale has also been used to evaluate pre-stroke disability levels (10). Pre-stroke mRS has been used extensively in research, audit, and service planning. For example, patients were excluded from seminal trials of acute stroke therapy if pre-stroke mRS was greater than a certain threshold (3, 4). In the UK national stroke audit, pre-stroke mRS is one of the core descriptors collected (11). In practice, decisions on treatment are often based on premorbid function (12).

Use of pre-stroke mRS for these purposes is potentially problematic as the clinical properties of the pre-stroke mRS have not been as thoroughly investigated as traditional poststroke mRS (13). The wording of the various levels of the scale, and the criteria used to distinguish one mRS grade from another, assume a previous stroke. This can cause issues in application when the scale is used to measure function before a stroke event. These issues are particularly problematic at lower mRS values, where comparison is made to the pre-stroke state. Although there is guidance on pre-stroke mRS scoring in those with a previous stroke event (14, 15), there is no internationally accepted consensus on how to use mRS as a pre-stroke measure in patients who have never had a previous stroke (14, 16).

Based on the current usage of pre-stroke mRS, key questions emerge. If pre-stroke mRS is used to assess prevalent disability, is it a valid measure of this construct? If pre-stroke disability is used as a component item in prognostic models, what is the independent contribution of pre-stroke mRS to outcome? If pre-stroke mRS is associated with outcome, can this be explained by differing process of care?

We aimed to use UK multicentre, clinical cohort to answer the above important and relevant research questions. Therefore, the specific aims of this study were:

(1) To assess the “validity” of pre-stroke mRS by comparison with other related disability metrics.
(2) To assess association of pre-stroke mRS with short and longer term mortality prognosis in a “real world” sample.
(3) To assess if pre-stroke mRS is associated with a differing process of care.

## Materials and Methods

### Population

We used the data held in the Anglia Stroke Clinical Network Evaluation Study (ASCNES). ASCNES was a multi-center, prospective cohort study. ASCNES collected clinical data from sequential stroke admissions across eight acute NHS (National Health Service) trusts in the East of England, UK (Norfolk, Suffolk, and Cambridgeshire). Data collection was from October 2009 to September 2011 inclusive. Data capture included a 1 year follow-up.

The full details of ASCNES have been described previously (17). In brief, included patients were aged over 18 years, with stroke confirmed and phenotyped by expert multidisciplinary clinical assessment. Our population included both first ever stroke and recurrent stroke and all included patients were treated as per institutional practice and stroke guidelines. Relevant institutional and ethical approvals for use of these data were in place.

### mRS Assessment

The ASCNES dataset was based on the modification of the Basic European Stroke Register Database but including process of care measures. Data were collected by clinical staff and transferred to an electronic database. Pre-stroke mRS was part of the initial assessment. All mRS assessments (pre- and poststroke) were performed by clinical staff using an unstructured interview and based on history taken from patient whenever possible, or their significant others/carers. The participating sites offered no explicit guidance on applying mRS grades as a pre-stroke measure and final score was at the discretion of the assessor.

### Analyses

We used basic descriptive statistics to describe baseline variables of included patients. As pre-stroke mRS was a key variable, we compared those with and without pre-stroke mRS against pre-specified variables of age, sex, NIHSS, stroke type (ischemic or haemorrhagic), systolic blood pressure, atrial fibrillation (AF), blood glucose, and Oxford Community Stroke Project (OCSP) classification.

### Validity of Pre-Stroke mRS

We described concurrent validity of pre-stroke mRS by comparison with other baseline clinical and demographic variables that are known to be associated with physical function. Our chosen comparators were, age, comorbidity burden assessed by Charlson comorbidity index (18), mRS at discharge, pre-stroke residence (categorized as: home, sheltered housing, rehabilitation center, care home), and receipt of formal care pre-stroke (categorized as: lives alone, lives with family, external carers, sheltered housing, institutional care).

We described association of pre-stroke mRS with other variables using chi-square for proportional data and Spearman rank correlation for nominal data. We re-categorized pre-stroke residence as “own home” or other (comprising any form of institutional care) and calculated odds-ratios for each pre-stroke mRS grade.

### Association of Pre-Stroke mRS and Outcomes

We examined the association between pre-stroke mRS and selected outcomes, which included mortality at 7 days and 1 year, length of stay (days), discharge destination (categorized as per our validity analyses), and poststroke complications of pneumonia and urinary tract infection. Due to modest numbers in pre-stroke mRS 4 and mRS 5, these categories were combined. We calculated univariable associations between pre-stroke mRS and outcomes of interest with strength of association described as odds ratio (OR) or beta for length of stay data. To compare pre-stroke mRS with other variables known to have prognostic importance, we also described association with outcomes for age, sex, stroke type, modified early warning score, glucose, AF, and comorbidity. We then calculated OR for pre-stroke mRS adjusted for other important prognostic variables (NIHSS, age, sex).

### Association of Pre-Stroke mRS and Process of Care

To describe association between pre-stroke mRS and process of care, we selected three aspects of acute stroke care that should be standard, are recommended in guidelines, and have been shown to have utility regardless of pre-stroke function. Our chosen process of care markers were: assessment of swallow (in first 24 h) (19); admission to dedicated stroke unit (SU) (days to SU admission from hospital admission and dichotomous yes/no) (20); brain imaging (days from admission to imaging and dichotomous yes/no) (21). Association of swallow test performed; admission to SU, and imaging performed with pre-stroke mRS was described for each factor using Mann–Whitney, association with categorized time to SU admission (days) and time to imaging (days) was described using Cuzik test for trend.

### Subgroup Analyses

Recognizing the difficulty in applying mRS to a population with no history of stroke, we performed subgroup analyses, we described our validity and prognostic analyses comparing results for those with a previous history of stroke against first ever strokes. Recognizing that the wording of the lower mRS grades make them more difficult to use as a pre-stroke assessment, we performed a further subgroup analysis comparing those with pre-stroke mRS 0–2 to those with pre-stroke mRS 3–5. Our dataset included patients admitted to University Teaching Hospitals and smaller “General” Hospitals. Sites could plausibly assess pre-stroke mRS differently depending on training, exposure to research, and staff mix. We tabulated pre-stroke mRS stratified by treating center.

## Results

A total of 2,491 patients with stroke were included in the ASCNES dataset. The mean age was 76.4 years (SD, 13.1), 1,311 (53%) were females; 2,120 (85%) had ischemic stroke (Table 1).

**Table 1:** Summary descriptive statistics of Anglia Stroke Clinical Network Evaluation Study data.

Variable	*N*	Mean (SD)/median (IQR)	Min	Max
Age (years)	2,487	76.4 (13.1)	18	101
Pre-stroke Rankin	2,001	0 (0–2)	0 (*N* = 1,027; 51.3%)	5 (*N* = 56; 2.7%)
MEWS^a^	2,101	1.4 (1.3)	0	7
NIHSS^a^	673	7 (3–14)	0	36
Systolic BP (mmHg)^a^	2,406	157.2 (30.5)	60	271
Glucose (mmol/l)^a^	2,136	7.6 (3.0)	1.4	40.2
	*N*	%		
**AF**				
No	1,469	59.0		
Yes	714	28.7		
Missing	308	12.4		
**Stroke type**				
ICH	320	85.1		
Infarct	2,120	12.9		
Missing	51	2.0		
**Bamford**				
LACS	520	20.9		
PACS	856	34.4		
POCS	308	12.4		
TACS	473	19.0		
Missing	334	13.4		
**Sex**				
Female	1,311	52.6		
Male	1,178	47.3		

*MEWS, modified early warning score (scale of 0–6+; higher scores indicate greater concern for health based on cardinal vital signs; 1–3 = low concern; 4 or 5 = medium concern; 6+ = high concern); NIHSS, National Institute of Health Stroke Scale; BP, blood pressure; AF, atrial fibrillation; ICH, intracerebral hemorrhage*.

*^a^Measured on admission*.

Pre-stroke mRS data were available for 2,001 patients. Median pre-stroke mRS was 0 (IQR, 0–2; range: 0–5) (Table 2). Pre-stroke mRS data were missing for 488 (24.4%) of the sample. There was no difference between those with and without mRS data for any of our pre-specified variables, other than OCSP where those with more severe strokes (TACS) were more likely to have missing pre-stroke mRS data (Supplementary Material). Data were also missing for those with AF (308; 12.4%), stroke type (51; 2%), and Bamford classification (334; 13.4%).

**Table 2:** Frequency of pre-stroke mRS score.

Pre-stroke mRS rating	Score frequency (%)
mRS 0	1,027 (51%)
mRS 1	367 (18%)
mRS 2	207 (10%)
mRS 3	212 (11%)
mRS 4	132 (7%)
mRS 5	56 (3%)

*mRS, modified Rankin scale*.

### Validity of Pre-Stroke mRS

Of included patients, 1,240 (49.8%) were living alone with no carers prior to admission. Median Charlson comorbidity index was 5 (IQR, 4–6). Age at time of stroke, discharge mRS, pre-stroke residence, pre-stroke care, and Charlson comorbidity index were all associated with pre-stroke mRS (rho >0.40 for continuous variables, *p* < 0.0001) (Table 3). Every point increase in pre-stroke mRS was associated with an increased association of living in institutional care pre-stroke. For example, comparing pre-stroke mRS 0 and 5, OR of institutional care pre-stroke was 73 [95% confidence interval (95% CI): 37–143] (Supplementary Material).

**Table 3:** Association of pre-stroke Rankin and other markers of function.

Factor	Association	*p*-Value
Age	0.40 (0.36, 0.44)^a^	<0.0001
mRs on discharge	0.50 (0.46, 0.53)^a^	<0.0001
Pre-stroke residence	995.52	<0.0001
Pre-stroke formal care received	761.12	<0.0001
Charlson comorbidity index	0.41 (0.37, 0.44)^a^	<0.0001

*^a^Spearman rank correlation coefficient (95% CI); two chi-squared test (DOF = 9)*.

*mRS, modified Rankin scale*.

On our subgroup analyses looking at those with previous stroke and those with lower pre-stroke mRS values, there was no clear and consistent difference between groups. Inspection of pre-stroke mRS classification by treating center suggested differential scoring at lower mRS grades between sites. The large number of sites and modest number of participants in certain grades precluded formal comparative analyses (Supplementary Material).

### Association of Pre-Stroke mRS and Outcomes

Mean length of stay was 16 days (SD, 20). Discharge to home, with no carers was recorded in 689 (28%); 400 (16%) were transferred from acute hospital to a rehabilitation facility. One year mortality was 770 (31%); 7-day mortality 265 (11%). Of poststroke complications, 288 (12%) developed pneumonia and 146 (6%) urinary tract infection. Every point increase in pre-stroke mRS was associated with greater numbers of poor outcomes for all our chosen outcomes. Strength of association was comparable to or higher than other known prognostic variables. Associations held when corrected for other covariates. For example, pre-stroke mRS score of 4 or 5 OR: 6.84 (95% CI: 4.24–11.03) compared to 0 in adjusted model [Table 4 (1-year mortality); Supplementary Material other outcomes].

**Table 4:** Association between factors and death within 1 year.

	Unadjusted		Adjusted	
	Odds ration (OR) (95% CI)	*p*-Value	OR (95% CI)	*p*-Value
Age (by year)	1.08 (1.07, 1.09)	<0.001	1.04 (1.02, 1.06)	<0.001
Male	0.65 (0.55, 0.77)	<0.001	0.79 (0.6, 1.06)	0.111
Pre-stroke Rankin		Trend: <0.001		Trend: <0.001
1 vs 0	2.23 (1.69, 2.94)	<0.001	1.88 (1.29, 2.74)	0.001
2 vs 0	2.82 (2.03, 3.92)	<0.001	1.85 (1.2, 2.85)	0.005
3 vs 0	5.48 (3.99, 7.51)	<0.001	3.43 (2.24, 5.25)	<0.001
4 or 5 vs 0	11.97 (8.4, 17.04)	<0.001	6.84 (4.24, 11.03)	<0.001
ICH	2.28 (1.79, 2.89)	<0.001	3.99 (2.69, 5.9)	<0.001
MEWS	1.32 (1.23, 1.41)	<0.001	1.18 (1.07, 1.31)	0.001
Glucose	1.07 (1.04, 1.10)	<0.001	1.06 (1.02, 1.11)	0.008
AF	2.08 (1.73, 2.52)	<0.001	1.59 (1.2, 2.12)	0.001
Charlson index	1.37 (1.32, 1.43)	<0.001	1.17 (1.08, 1.26)	<0.001

*ICH, intracerebral hemorrhage; MEWS, Modified Early Warning Score; AF, atrial fibrillation*.

On our subgroup analyses looking at those with previous stroke and those with lower pre-stroke mRS values, there was no clear and consistent difference between groups (Supplementary Material).

### Pre-Stroke mRS and Processes of Care

Of 2,383 patients with relevant data, 1,674 (70%) had a swallow test performed; 2,287 (96%) had brain imaging, of whom 1,520 (64%) were scanned within 24 h; and 1,886 (79%) were admitted to a SU of whom 1,162 (49%) were admitted on the same day. There was a difference in pre-stroke mRS between those who received swallow assessment (*p* = 0.04) and brain imaging (*p* = 0.0003), but not admission to ASU (*p* = 0.21). This difference favored those with greater disability, i.e., higher pre-stroke mRS was more likely to receive these evidence-based aspects of care. There was no apparent difference in time to SU admission or imaging by pre-stroke mRS.

## Discussion

We sought to assess some of the key clinical properties of the pre-stroke mRS in order to address important questions as to its use in clinical research and practice. According to our results, the pre-stroke mRS has a validity that is close to what is considered as fair (<0.5) (20), albeit in the absence of a true gold standard for pre-stroke function, these analyses are open to interpretation and the correlation described may be acceptable. Pre-stroke mSR is a potential tool for predicting prognostic outcome following stroke and the relationship between pre-stroke mRS scores and prognosis is not related to variations in patient care pathways.

Arguably, the most important property of pre-stroke mRS is validity; the scale is of little utility if it does not measure what it purports to measure. We assessed concurrent validity and found that the pre-stroke mRS demonstrated significant associations with all our chosen measures that should reflect pre-stroke disability. Although significant, the correlation we demonstrated was at best moderate in strength. This finding is similar to that of Fearon (6) who found moderate correlations between pre-stroke mRS and pre-stroke comorbidity and frailty, but not pre-stroke care needs. Although there are concerns that pre-stroke mRS may not be suited to first stroke events or those with good function pre-stroke, our subgroup analyses did not find any convincing evidence of this. Our results should reassure clinicians and researchers that pre-stroke mRS does capture pre-stroke function, but the moderate associations suggest that there may be scope for further improvement.

Our results demonstrate that the pre-stroke mRS is a potential prognostic indicator. Although pre-stroke mRS is widely accepted to be an indicator of prognosis, there has been very little published work to confirm this. We found that as the pre-stroke mRS score increased, patients were more likely to experience negative outcomes, and this pattern was consistent across all of our chosen outcome measures (LOS, discharge destination, mortality, and complications after stroke). Furthermore, the strength of association of the pre-stroke mRS with each outcome variable was comparable or greater than that of all of our other prognostic variables, suggesting that the prognostic utility of pre-stroke mRS is at least as strong as other commonly used indicators of prognosis. These findings are broadly consistent with those of Kwok et al. (5) who also found that pre-stroke mRS was a robust predictor of poststroke outcome, as measured by mortality and length of stay. Our findings also extend the prognostic predictability of the pre-stroke mRS to that of eventual discharge destination and the prevalence of complications poststroke.

Importantly, we also found that the association between pre-stroke mRS and outcome cannot be explained by differing process of care. Specifically, our results reveal that while there is a difference in the process of stroke care associated with pre-stroke mRS, this difference indicated that individuals who had higher pre-stroke mRS scores were more likely to be provided with access to evidence-based stroke care. For instance, significantly more patients with higher pre-stroke disability scores received a swallow assessment and brain imaging; while there was no difference found in regards to pre-stroke mRS and time taken for admission to a SU or for brain imaging. If anything, the pattern of care is likely to have reduced the association between pre-stroke mRS and outcome. It is, therefore, reassuring that the relationship between pre-stroke mRS and outcome appears to be based purely on the effect of premorbid disability on the overall impact of the stroke. Our data did not allow for more sophisticated analyses looking at hospital level confounders such as teaching hospital vs non-teaching hospital or staffing levels.

While our results suggest that the pre-stroke mRS has moderate validity as a tool for measuring pre-stroke function, more work is required on this property. Confidence in the ability of the pre-stroke mRS to detect premorbid disability is low and Bruno and Switzer (22) have gone as far as to say that the pre-stroke mRS is not fit for purpose in this regard. Future studies that focus on comparing the pre-stroke mRS with a more detailed assessment of pre-stroke function would help to resolve this question of validity.

Our study had a number of strengths. We had a large participant pool that allowed a multitude of comparisons between variables enabling revealing insights into the clinical properties of pre-stroke mRS. Our sample was also taken from a “real world” patient pool of consecutive, unselected stroke admissions, providing good generalizability of our results to the wider population.

However, we acknowledge some limitations of our study. Most notably, around 20% of our sample did not have pre-stroke mRS scores. Further analyses revealed that there were no significant differences of characteristics in the missing-data group compared with the data-intact group with the exception of stroke-type. Missing mRS data were more common for the severe stroke types (TACS and PACS). For a clinical registry, this pattern of missing data is not surprising. The more severe strokes will be less able to engage in assessments of pre-stroke status. Although these missing data create a potential bias, the effect is likely to have reduced the associations between pre-stroke mRS and outcome, rather than exaggerated it. Furthermore, the internal relationship and effect sizes observed between pre-stroke mRS and other measures are not affected by missing data. We acknowledge that we have no detail on how pre-stroke mRS was applied at each site. In the absence of explicit guidance on scoring, there is the potential for inter-rater variability in pre-stroke mRS. However, potential for variation in scoring is an issue for mRS *per se* (16), and our data will reflect the way in which pre-stroke mRS is currently used in clinical practice, giving our results “real world” validity. Finally, our validation analysis is limited by an imperfect reference standard. Constructs such as place of residence are a blunt indicator of functional status.

In our analysis of the pre-stroke state, we were limited to those assessments that had been routinely collected in our dataset. We did not have comprehensive data on potentially important covariates such as NIHSS. There is no single screening tool that will perfectly describe global functional ability. Assessments scales such as the Informant Questionnaire for Cognitive Decline in the Elderly (23) and Barthel Index (24) have been used to capture pre-stroke cognitive and physical ability, and it is unfortunate that these data were not routinely collected during the period of our study. Nonetheless, in the absence of a clear “gold standard” of pre-stroke function, the correlations with all of our measures in combination provides us with confidence in our validation method.

Based on our findings, we offer guidance for clinicians and researchers. As a standard mRS is only a moderately valid measure of pre-stroke function, we would caution against using this as the sole criterion for assessing suitability for research or for interventions. To try and improve consistency, we would recommend following published guidance and best practice in assessment (14). A more comprehensive assessment of pre-stroke function could include measures of ability to perform activities of daily living and cognitive function. These assessments need not necessarily add substantial time to assessment, for example, a short-form Barthel Index for use in stroke has been described (25). We would encourage greater use of pre-stroke measures in clinical practice, our data on the prognostic utility of mRS remind us of the importance of considering the pre-stroke state.

We recognize plausible methods to improve the validity of pre-stroke assessment, and future research could focus on these areas. If we continue to use mRS, there may be scope to further operationalize the assessment. Structured questionnaire approaches to mRS have been described (26) and a similar approach to pre-stroke mRS could have utility. As mRS was never designed as a tool to measure pre-stroke function, perhaps we should move to a more relevant measure. There is increasing interest in tools to describe frailty (27) and these may have particular utility in an older adult stroke cohort.

## Conclusion

In summary, we have assessed the prognostic predictability of the pre-stroke mRS and its validity as a measure of pre-stroke function. In combination, these results highlight that the pre-stroke mRS can reliably be employed as a tool to assist clinicians in service delivery and planning. We found that the pre-stroke mRS is a moderately valid measure of pre-stroke disability and a robust predictor of poststroke prognosis. In combination, these results highlight that the pre-stroke mRS can reliably be employed as a tool to assist clinicians in service delivery and planning. The robust nature of the relationship between pre-stroke mRS and a number of different outcomes suggests that the pre-stroke mRS not only give an insight into likely mortality or duration in hospital but may also predict the probability of institutional care that patients will need to be taken into care poststroke, as well as potential poststroke complications experienced during the recovery period. Importantly, the prognostic accuracy of the pre-stroke mRS does not appear to be related to any variation in the process of care. Thus, prognostic models of stroke, which incorporate pre-stroke mRS are likely to have potential for future clinical implication.

## Author Contributions

TQ and PM conceived the study. PM is the PI of the Anglia Stroke Clinical Network Evaluation Study. JP, MB, DD, AM, and EW are co-I of the ASCNES. SM was responsible for data management and AC analyzed the data. MT-R and TQ drafted the paper. All authors contributed to the writing of the paper. PM is the guarantor.

## Conflict of Interest Statement

The authors declare that the research was conducted in the absence of any commercial or financial relationships that could be construed as a potential conflict of interest.
