# Supplementary material for: Pre-Stroke Modified Rankin Scale: Evaluation of Validity, Prognostic Accuracy, and Association with Treatment
Source: Front Neurol. 2017 Jun 13;8:275. doi: 10.3389/fneur.2017.00275 (PMC5468801; doi:10.3389/fneur.2017.00275)
Supplement: Supplementary file 1 [file Data_Sheet_1.PDF]

Supplementary Material

Supplementary Table V. Association between factors and death on discharge.

	Unadjusted		Adjusted	
	OR (95% CI)	p-value	OR (95% CI)	p-value
Age	1.06 (1.05,1.07)	<0.001	1.04 (1.02,1.07)	<0.001
Male	0.67 (0.55,0.82)	<0.001	0.98 (0.69,1.38)	0.897
Pre-stroke rankin		Trend: <0.001		<0.001
1 vs 0	1.87 (1.32,2.64)	<0.001	1.72 (1.06,2.78)	0.028
2 vs 0	2.46 (1.66,3.65)	<0.001	2.24 (1.33,3.78)	0.002
3 vs 0	3.70 (2.57,5.32)	<0.001	2.76 (1.67,4.57)	<0.001
4 or 5 vs 0	8.25 (5.79,11.75)	<0.001	4.71 (2.84,7.79)	<0.001
ICH	3.20 (2.48,4.14)	<0.001	5.04 (3.34,7.61)	<0.001
MEWS	1.50 (1.39,1.62)	<0.001	1.38 (1.23,1.55)	<0.001
Glucose	1.08 (1.05,1.12)	<0.001	1.09 (1.03,1.14)	0.001
AF	1.81 (1.46,2.26)	<0.001	1.33 (0.95,1.87)	0.095
Charlson index	1.24 (1.19,1.29)	<0.001	1.07 (0.98,1.17)	0.118

ICH= Intracerebral haemorrhage; MEWS= Modified early warning system; AF= Atrial fibrilation

Supplementary Table VI. Association between factors and death within 7 days.

	Unadjusted		Adjusted	
	OR (95% CI)	p-value	OR (95% CI)	p-value
Age	1.04 (1.03,1.05)	<0.001	1.02 (1,1.04)	0.11
Male	0.77 (0.59,0.99)	0.045	1.01 (0.64,1.58)	0.98
Pre-stroke rankin		Trend:<0.001		Trend:<0.001
1 vs 0	1.62 (1.04,2.50)	0.031	1.83 (0.97,3.44)	0.06
2 vs 0	1.79 (1.06,3.01)	0.029	2.24 (1.11,4.5)	0.024
3 vs 0	2.12 (1.30,3.46)	0.003	2.54 (1.3,4.96)	0.006
4 or 5 vs 0	5.58 (3.68,8.46)	<0.001	4.39 (2.32,8.28)	<0.001
ICH	5.03 (3.75,6.75)	<0.001	8.22 (5.14,13.15)	<0.001
MEWS	1.68 (1.53,1.85)	<0.001	1.6 (1.38,1.84)	<0.001
Glucose	1.09 (1.05,1.13)	<0.001	1.06 (1,1.12)	0.035
AF	1.48 (1.12,1.95)	0.006	1.16 (0.74,1.81)	0.518
Charlson index	1.16 (1.10,1.22)	<0.001	1.09 (0.97,1.21)	0.137

ICH= Intracerebral haemorrhage; MEWS= Modified early warning system; AF= Atrial fibrilation

Supplementary Table VII. Association between factors and UTI.

	Unadjusted		Adjusted	
	OR (95% CI)	p-value	OR (95% CI)	p-value
Age	1.02 (1.01,1.04)	0.005	1.04 (1.02,1.07)	0.002
Male	0.58 (0.41,0.82)	0.002	0.6 (0.37,0.97)	0.036
Pre-stroke rankin		Trend: 0.090		Trend: 0.231
1 vs 0	2.32 (1.44,3.72)	<0.001	2.18 (1.2,3.96)	0.01
2 vs 0	2.93 (1.72,4.99)	<0.001	3.42 (1.79,6.53)	<0.001
3 vs 0	1.79 (0.97,3.28)	0.062	1.47 (0.68,3.16)	0.323
4 or 5 vs 0	1.04 (0.48,2.26)	0.916	1.21 (0.49,2.96)	0.682
ICH	0.93 (0.56,1.54)	0.772	0.96 (0.46,2)	0.922
MEWS	0.89 (0.77,1.03)	0.109	0.86 (0.71,1.03)	0.097
Glucose	0.99 (0.93,1.05)	0.674	0.97 (0.89,1.06)	0.552
AF	1.57 (1.10,2.26)	0.014	1.48 (0.93,2.35)	0.097
Charlson index	1.02 (0.95,1.09)	0.683	0.77 (0.64,0.92)	0.004

ICH= Intracerebral haemorrhage; MEWS= Modified early warning system; AF= Atrial fibrilation

Supplementary Table VIII. Association between factors and pneumonia.

	Unadjusted		Adjusted	
	OR (95% CI)	p-value	OR (95% CI)	p-value
Age	1.04 (1.03,1.06)	<0.001	1.04 (1.02,1.07)	<0.001
Male	0.86 (0.67,1.11)	0.243	1.09 (0.75,1.6)	0.639
Pre-stroke rankin		Trend: 0.001		Trend: 0.001
1 vs 0	1.56 (1.02,2.38)	0.04	1.32 (0.78,2.23)	0.307
2 vs 0	2.06 (1.27,3.33)	0.003	1.67 (0.94,2.98)	0.081
3 vs 0	2.74 (1.76,4.26)	<0.001	1.53 (0.86,2.75)	0.15
4 or 5 vs 0	4.12 (2.7,6.29)	<0.001	2.79 (1.62,4.82)	<0.001
ICH	1.09 (0.76,1.56)	0.642	1.24 (0.74,2.08)	0.416
MEWS	1.31 (1.20,1.43)	<0.001	1.18 (1.04,1.34)	0.012
Glucose	1.06 (1.02,1.10)	0.002	1.06 (1.01,1.11)	0.025
AF	1.61 (1.24,2.11)	<0.001	1.38 (0.96,1.99)	0.086
Charlson index	1.14 (1.09,1.20)	<0.001	0.96 (0.86,1.06)	0.425

ICH= Intracerebral haemorrhage; MEWS= Modified early warning system; AF= Atrial fibrilation

Supplementary Table IX. Association between factors and length of stay.

	Unadjusted		Adjusted	
	Beta (95% CI)	p-value	Beta (95% CI)	p-value
Age	0.26 (0.20,0.32)	<0.001	0.2 (0.09,0.31)	<0.001
Male	-3.28 (-4.85,-1.71)	<0.001	-1.56 (-3.72,0.59)	0.155
Pre-stroke rankin		Trend: <0.001		
1 vs 0	6.01 (3.78,8.24)	<0.001	5.68 (2.71,8.65)	<0.001
2 vs 0	6.11 (3.32,8.9)	<0.001	4.39 (0.81,7.96)	0.016
3 vs 0	9.28 (6.51,12.05)	<0.001	6.38 (2.77,10)	0.001
4 or 5 vs 0	3.58 (0.68,6.49)	0.016	1.06 (-2.87,4.98)	0.597
ICH	1.52 (-0.83,3.87)	0.204	2.79 (-0.4,5.97)	0.086
MEWS	0.01 (-0.62,0.64)	0.971	-0.15 (-0.96,0.65)	0.707
Glucose	0.19 (-0.10,0.48)	0.192	0.18 (-0.17,0.52)	0.313
AF	5.53 (3.77,7.30)	<0.001	4.11 (1.83,6.4)	<0.001
Charlson index	0.85 (0.51,1.19)	<0.001	-0.38 (-0.98,0.22)	0.219

Supplementary Table X. Association between factors and 'poor outcome' for those at home.

	Unadjusted		Adjusted	
	OR (95% CI)	p-value	OR (95% CI)	p-value
Age	1.04 (1.03,1.05)	<0.001	1.03 (1.02,1.05)	<0.001
Male	0.59 (0.49,0.70)	<0.001	0.64 (0.49,0.83)	0.001
Pre-stroke rankin		Trend: <0.001		Trend: <0.001
1 vs 0	1.81 (1.41,2.33)	<0.001	2 (1.42,2.83)	<0.001
2 vs 0	2.17 (1.57,3)	<0.001	2.25 (1.47,3.45)	<0.001
3 vs 0	3.13 (2.15,4.57)	<0.001	2.62 (1.61,4.28)	<0.001
4 or 5 vs 0	3.61 (2.28,5.73)	<0.001	2.24 (1.2,4.19)	0.011
ICH	2.73 (2.10,3.56)	<0.001	3.7 (2.47,5.54)	<0.001
MEWS	1.24 (1.16,1.34)	<0.001	1.17 (1.06,1.29)	0.002
Glucose	1.08 (1.04,1.11)	<0.001	1.06 (1.01,1.11)	0.011
AF	1.95 (1.60,2.38)	<0.001	1.69 (1.28,2.24)	<0.001
Charlson index	1.16 (1.12,1.21)	<0.001	0.98 (0.91,1.06)	0.614

Table XI: Association of Pre-stroke Rankin and other markers of function

	All	No Prev-stroke	Prev-stroke	mRS 0-2	mRs 3-5
Factor	Association				
Age	0.40 (0.36,0.44)[1]	0.42 (0.38,0.46)	0.24 (0.15,0.33)	0.30 (0.25,0.34)	0.00 *(-0.09,0.10)
mRs on discharge	0.50 (0.46,0.53)[1]	0.47 (0.43,0.51)	0.54 (0.47,0.61)	0.32 (0.27,0.36)	0.34 (0.25,0.42)
Pre-stroke residence	995.5[2]	803.2	216.4	95.5	63.7
Pre-stroke formal care received	761.1[2]	649.0	145.2	181.8	40.9
Charlston co morbidity index	0.41 (0.37,0.44)[1]	0.40 (0.36,0.44)	0.21 (0.12,0.30)	0.33 (0.29,0.38)	0.06 *(-0.04,0.16)

[1] Spearman rank correlation; [2] Chi-squared test. All values $p<0.0001$ unless indicated by*.

Table XIIa: Association between factors and death within 1 year (by previous stroke).

	No previous stroke				Previous stroke			
	Unadjusted		Adjusted		Unadjusted		Adjusted	
	OR (95% CI)	p-value	OR (95% CI)	p-value	OR (95% CI)	p-value	OR (95% CI)	p-value
Age	1.08 (1.07,1.09)	<0.001	1.04 (1.02,1.06)	0	1.06 (1.04,1.09)	<0.001	1.05 (1.01,1.09)	0.011
Male	0.61 (0.5,0.74)	<0.001	0.77 (0.55,1.07)	0.117	0.78 (0.55,1.12)	0.187	0.87 (0.48,1.56)	0.635
Pre-stroke rankin		Trend:<0.001		Trend:<0.001		Trend:<0.001		Trend:<0.001
1 vs 0	1.96 (1.43,2.69)		1.58 (1.03,2.43)		3.15 (1.69,5.85)		3.86 (1.62,9.21)	
2 vs 0	2.74 (1.86,4.05)		1.77 (1.06,2.95)		2.82 (1.45,5.48)		2.52 (1.06,5.99)	
3 vs 0	5.82 (4.02,8.43)		3.56 (2.12,5.99)		4.48 (2.33,8.65)		4.14 (1.79,9.58)	
4or5 vs 0	12.72 (8.13,19.9)		8.6 (4.61,16.04)		10.21 (5.31,19.64)		6.64 (2.81,15.67)	
Bleed	2.32 (1.77,3.03)	<0.001	3.75 (2.39,5.89)	0	2.2 (1.29,3.76)	0.004	5.22 (2.27,11.98)	0
MEWS	1.32 (1.22,1.43)	<0.001	1.18 (1.05,1.33)	0.006	1.3 (1.13,1.49)	<0.001	1.18 (0.97,1.45)	0.104
Glucose	1.08 (1.04,1.11)	<0.001	1.06 (1.01,1.12)	0.027	1.06 (0.98,1.14)	0.136	1.07 (0.97,1.18)	0.173
AF	2.1 (1.69,2.61)	<0.001	1.59 (1.14,2.22)	0.007	1.88 (1.28,2.77)	0.001	1.7 (0.97,2.99)	0.065
Charlson index	1.39 (1.32,1.46)	<0.001	1.19 (1.1,1.3)	0	1.28 (1.16,1.41)	<0.001	1.15 (0.97,1.36)	0.1

OR= Odds Ratio; AF= Atrial Fibrilation; MEWS= Modified Early Warning Score

Table XIIb: Association between factors and death within 1 year (by mRS subgroup).

	mRs 0-2				mRs 3-5			
	Unadjusted		Adjusted		Unadjusted		Adjusted	
	OR (95% CI)	p-value	OR (95% CI)	p-value	OR (95% CI)	p-value	OR (95% CI)	p-value
Age	1.07 (1.05,1.08)	<0.001	1.03 (1.01,1.05)	0.001	1.05 (1.03,1.07)	<0.001	1.06 (1.02,1.1)	0.001
Male	0.71 (0.56,0.9)	0.004	0.79 (0.57,1.09)	0.153	0.79 (0.52,1.2)	0.272	0.78 (0.42,1.45)	0.436
Pre-stroke rankin		Trend:<0.001		Trend:<0.001				
1 vs 0	2.23 (1.69,2.94)		1.91 (1.31,2.8)	0.001				
2 vs 0	2.82 (2.03,3.92)		1.84 (1.19,2.84)	0.006				
4or5 vs 3					2.19 (1.45,3.3)	<0.001	2.07 (1.18,3.63)	0.011
Bleed	2.79 (2.05,3.8)	<0.001	4.5 (2.93,6.91)	0	1.92 (0.98,3.77)	0.058	2.28 (0.93,5.55)	0.07
MEWS	1.3 (1.19,1.43)	<0.001	1.21 (1.07,1.36)	0.002	1.19 (1.02,1.4)	0.027	1.11 (0.91,1.35)	0.305
Glucose	1.07 (1.03,1.12)	0.001	1.04 (0.99,1.1)	0.103	1.11 (1.01,1.22)	0.023	1.17 (1.04,1.32)	0.01
AF	1.88 (1.44,2.44)	<0.001	1.43 (1.02,1.99)	0.038	1.98 (1.27,3.1)	0.003	2.22 (1.26,3.93)	0.006
Charlson index	1.33 (1.26,1.41)	<0.001	1.22 (1.12,1.33)	0	1.18 (1.07,1.31)	0.001	1.05 (0.91,1.21)	0.493

OR= Odds Ratio; AF= Atrial Fibrilation; MEWS= Modified Early Warning Score

Table XIII Pre-stroke mRS scoring by centre contributing data

	Unit 1	Unit 2	Unit 3	Unit 4	Unit 5	Unit 6	Unit 7	Unit 8
mRS 0	99 (24.6)	5 (23.8)	128 (34.5)		368 (52.6)	148 (46.3)	153 (54.8)	126 (50.8)
mRS 1	68 (16.9)	5 (23.8)	76 (20.5)		97 (13.9)	17 (5.3)	65 (23.3)	39 (15.7)
mRS 2	26 (6.4)	4 (19.1)	55 (14.8)		61 (8.7)	17 (5.3)	20 (7.2)	24 (9.7)
mRS 3	24 (6.0)	2 (9.5)	39 (10.5)		76 (10.9)	22 (6.9)	16 (5.7)	33 (13.3)
mRS 4	9 (2.2)	4 (19.1)	39 (10.5)		38 (5.4)	12 (3.8)	10 (3.6)	20 (8.1)
mRS 5	0 (0.0	1 (4.8)	8 (2.2)		26 (3.7)	9 (2.8)	6 (2.2)	6 (2.4)
missing	176 (43.8)	0 (0.0)	26 (7.0)	150 (100)	34 (4.9)	95 (29.7)	9 (3.2)	0 (0.0

Data are n (%) for each centre that contributed data to the database, unit 4 did not contribute pre-stroke mRS data.
